# Cytoplasmic eIF6 promotes OSCC malignant behavior through AKT pathway

**DOI:** 10.1186/s12964-021-00800-4

**Published:** 2021-12-18

**Authors:** Zechen Zhao, Weiming Chu, Yang Zheng, Chao Wang, Yuemei Yang, Teng Xu, Xueming Yang, Wei Zhang, Xu Ding, Gang Li, Hongchuang Zhang, Junbo Zhou, Jinhai Ye, Heming Wu, Xiaomeng Song, Yunong Wu

**Affiliations:** 1 Jiangsu Key Laboratory of Oral Diseases, Nanjing Medical University, No.1, Shanghai Road, Gulou District, Nanjing, Jiangsu 210029 People’s Republic of China; 2 Department of Oral and Maxillofacial Surgery, Affiliated Hospital of Stomatology, Nanjing Medical University, Nanjing, Jiangsu People’s Republic of China; 3 Jiangsu Province Engineering Research Center of Stomatological Translational Medicine, Nanjing Medical University, Nanjing, Jiangsu People’s Republic of China; 4 Department of Stomatology, Clinical Medical College, Yangzhou University, Yangzhou, Jiangsu People’s Republic of China; 5 Department of Oral Maxillofacial and Head and Neck Oncology, Shanghai Ninth People’s Hospital Affiliated to Shanghai Jiao Tong University School of Medicine, National Clinical Research Center for Oral Disease, National Center of Stomatology, Shanghai, 200011 China; 6 Department of Stomatology, The Affiliated People’s Hospital of Jiangsu University, Zhenjiang, Jiangsu People’s Republic of China; 7 Department of Stomatology, Affiliated Hospital of Xuzhou Medical University, Xuzhou, Jiangsu People’s Republic of China; 8 Department of Stomatology, Xuzhou No.1 Peoples Hospital, Xuzhou, Jiangsu People’s Republic of China; 9 Department of Stomatology, Nanjing Integrated Traditional Chinese and Western Medicine Hospital, Nanjing, Jiangsu People’s Republic of China

**Keywords:** OSCC, eIF6, EMT, AKT, Cell invasion and migration

## Abstract

**Background:**

Eukaryotic translation initiation factor 6 (eIF6), also known as integrin β4 binding protein, is involved in ribosome formation and mRNA translation, acting as an anti-association factor. It is also essential for the growth and reproduction of cells, including tumor cells. Yet, its role in oral squamous cell carcinoma (OSCC) remains unclear.

**Methods:**

The expression characteristics of eIF6 in 233 samples were comprehensively analyzed by immunohistochemical staining (IHC). Effects of eIF6 over-expression and knockdown on cell proliferation, migration and invasion were determined by CCK-8, wound healing and Transwell assays. Western blot, immunofluorescence (IF) and co-immunoprecipitation (co-IP) were performed for mechanical verification.

**Results:**

We found that cytoplasmic eIF6 was abnormally highly expressed in OSCC tissues, and its expression was associated with tumor size and the clinical grade. Amplification of eIF6 promoted the growth, migration and invasion capabilities of OSCC cell lines in vitro and tumor growth in vivo*.* Through Western blot analysis, we further discovered that eIF6 significantly promotes epithelial-mesenchymal transformation (EMT) in OSCC cells, while depletion of eIF6 can reverse this process. Mechanistically, eIF6 promoted tumor progression by activating the AKT signaling pathway. By performing co-immunoprecipitation, we discovered a direct interaction between endogenous eIF6 and AKT protein in the cytoplasm.

**Conclusion:**

These results demonstrated that eIF6 could be a new therapeutic target in OSCC, thus providing a new basis for the prognosis of OSCC patients in the future.

**Video abstract**

**Supplementary Information:**

The online version contains supplementary material available at 10.1186/s12964-021-00800-4.

## Background

Head and neck cancer is the 6th most common cancer worldwide [1], causing nearly 700,000 new cases and 380,000 deaths every year [2]. The majority of these patients are diagnosed with head and neck squamous cell carcinoma, including oral squamous cell carcinoma (OSCC) [3]. Despite advances in surgery, radiotherapy, and chemotherapy, 40–60% of OSCC patients have local recurrence or distant metastasis [4, 5]. Therefore, the in-depth study of risk factors and molecular biomarkers has provided a new idea for subsequent treatment [6].

It is well known that ribosomes translate biological proteins, and the initial stage of this process is a pivotal step to regulate and limit translation [7]. The eukaryotic translation initiation factors have an indispensable role at this stage, such as eIF1, eIF3, eIF5, etc. [8]. eIF6, also known as integrin β4 binding protein, is mainly located in the cytoplasm of mammalian cells and partly in the nucleus. However, eIF6 is also located in individual cells’ nucleolus, like HeLa, A431, Colon adenoma, and cancer cell lines [9]. eIF6 can prevent the premature combination of the 40 s ribosomal subunit and the 60 s ribosomal subunit in the translation initiation stage, thus exerting its role in rate-limiting regulation [10]. eIF6 has a unique effect on the regulation of biological growth and development [11]. In Xenopus, over-expressing eIF6 can lead to delayed eye development [12]. Gipc2 regulated by eIF6 can ensure the Xenopus eye's normal morphology development and stimulate the relative molecular network, such as the AKT signaling pathway [13]. In mammals, eIF6 acts on adipogenic transcription factors, such as C/EBPb, C/EBPd, and ATF4, thereby affecting lipid metabolism and glycolysis level [14]. Given the powerful translation control capabilities of eIF6, we began to explore whether it continues to be important in cancer development.

The importance of eIFs in the development of tumors has been constantly evaluated. The carcinogenicity of multiple eIF subunits has been proven in various tumor entities[15]. According to reports, colorectal cancer cells with mutations or deletions of APC (adenomatous polyposis coli) gene are strongly dependent on eIF2B5. eIF2B5 restricts the expression of MYC (Myelocytomatosis viral oncogene homolog) to prevent apoptosis and promote tumor growth[16]. In ovarian cancer, eIF3C can be used as a direct target of YTHDF1 (YT521-B homology domain-containing Proteins F1). M^6^A-modified (N^6^-Methyladenosine) eIF3C can be combined with YTHDF1 to accelerate the process of overall mRNA translation, thereby promoting tumorigenesis and metastasis[17]. In addition, the FOXO3a (forkhead box O3A) /eIF4EBP1 (eIF4E binding protein 1) axis plays a critical role in the inhibitory effect of G129R(a prolactin antagonist) on cell proliferation and cell cycle in uterine cancer[18]. In terms of tumor drug resistance, overexpression of eIF3a might play a guiding role in the application of platinum-based chemotherapy[19].

According to previous studies, eIF6 is abnormally expressed in human cancer tissues [9]. In thyroid carcinoma, eIF6 promotes tumor growth by regulating MIR-144-3p/TGF-α, and the knockout of eIF6 enhances cisplatin sensitivity [20]. In non-small cell lung cancer, the expression of eIF6 is higher than in normal tissues. Knocking out eIF6 leads to pre-rRNA processing and 60 s ribosome maturation defects [21]. In addition, eIF6 is related to patients’ survival rate in colorectal cancer. Its absence can inhibit cell proliferation and invasion [22]. Besides, studies have suggested that eIF6 in tumors is related to its hyperphosphorylation [23]. When eIF6 takes effect, it remarkably upregulates the functional network related to cell movement, like the CDC22, which can significantly increase the migration and invasion of tumor cells [24].

During embryonic development, cell epithelium’s transition to mesenchymal state is a highly plastic and dynamic process called epithelial-mesenchymal transition (EMT) that allows migration and invasion behavior [25]. In malignant cells, EMT transcription factors, such as ZEB1(Zinc-finger E-box binding homeobox 1) and ZEB2 (Zinc finger E-box binding homeobox 2) can lead to E-cadherin transcriptional repression and a rise in N-cadherin and vimentin [26, 27]. According to reports, PERK-eIF2α (protein kinase RNA-like extracellular regulated protein kinase-eIF2) is signal transduction necessary to maintain endoplasmic reticulum homeostasis, which is also important for the invasion of EMT cells [28]. EIF5A2 promotes the invasion and metastasis of liver cancer by inducing EMT and activates RhoA and Rac1 (Rho-family small GTPases) to cause cytoskeletal rearrangement [29]. However, whether eIF6 can affect the full migration and invasion capabilities of OSCC remains unclear.

In this study, we found that cytoplasmic eIF6 might have a vital role in the progression of OSCC and mediate AKT-related signaling pathways. In OSCC, a high expression of eIF6 activated EMT to promote cell migration and invasion. On the other hand, the knockdown of eIF6 reversed this process. AKT pathway inhibitor, LY294002, reversed the oncogenic phenotype of eIF6 in OSCC. By performing Co-IP, we discovered a physical binding between endogenous eIF6 and AKT protein. Overall, these results demonstrated that eIF6 could be a new therapeutic target in OSCC, thus providing a new basis for the prognosis of OSCC patients in the future.

## Methods

### Patients and tissue samples

Tumor microarrays were assessed in this study. A total of 233 patient samples were collected from the Stomatological Hospital of Jiangsu Province (In 2014–2019), including 206 primary OSCC samples and 27 normal oral mucosae. Clinical and pathological data were listed in Table 1. Patient clinical information included age, gender, location, tumor size, histological grade, metastatic lymph node, clinical stage (defined by the American Joint Committee on Cancer 7th edition), and postoperative survival rate (as of April 2021). Besides, we collected 8 samples of tumor tissues and adjacent normal tissues from patients diagnosed with OSCC from the Stomatological Hospital of Jiangsu Province (In 2020). All samples were stored in -80℃ liquid nitrogen for subsequent experiments. Corresponding clinicopathological data are shown in Additional file 1: Table S1. This study was approved by the Ethics Committee of Nanjing Medical University. Informed consent was obtained from all patients.

**Table 1.** Correlation between eIF6 and clinicopathologic characteristics in 184 OSCC cases

Pathologic characteristics	n	Overexpression (number of cases)	Nonoverexpression (number of cases)	P value
Age, years				
≥ 60	98	53	45	0.1951
<60	86	46	40	
Sex				
Male	114	57	57	0.7918
Female	70	42	28	
Smoking				
Yes	76	42	34	0.2070
No	108	60	48	
Drinking				
Yes	90	49	41	0.2126
No	94	53	41	
Location				
Palate	10	4	6	0.7236
Tongue	63	33	30	
Gingiva	35	21	14	
Buccal	59	33	26	
Mouth floor	17	8	9	
Tumor stage				
T1	83	35	48	T1 vs T2 = 0.0123
T2	74	46	28	T1 vs T3–4 = 0.0013
T3–T4	27	21	6	
Lymph node status				
N0	99	39	60	N0 vs N1 = 0.0187
N1	39	24	15	N0 vs N2–3 <0.0001
N2–3	46	35	11	
Clinical grade				
I	49	20	29	I vs II–III = 0.0005
II–III	67	49	18	I vs IV <0.0001
IV	68	58	10	
Pathological grade				
I	107	58	49	I vs II = 0.9052
II	58	32	26	I vs III = 0.7406
III	18	9	9	

The P values represent probabilities for eIF6 expression levels between variable subgroups determined by a ^χ2^ test.

### Immunohistochemistry

Tissue microarrays were stained with primary antibodies against eIF6 (diluted 1:200, Abcam) overnight following secondary antibody incubation for 30 min. All of the sections were counterstained using hematoxylin, dehydrated, cleared and mounted before examination using a microscope (DM4000B, Leica, Germany). eIF6 immunoreactivity in microarray samples was calculated according to staining concentration and proportion semi-quantitatively. The score for the scale of positive cells was demonstrated as follows: 0, negative; 1, < 20%; 2, 20- 50%; 3, 51–75%; and 4, > 75% positive cells. For staining strength, grading system was classified as below: 0, no staining; 1, light yellow; 2, brownish yellow; 3, dark brownish yellow. The result was calculated by multiplying the two scores as mentioned above. Scores for > 4 points were regarded as positive.

### Cell culture and inhibitor

Human OSCC cell lines HN4 and HN6 were obtained from the Shanghai Ninth People's Hospital (Shanghai, China). Cells were cultured in Dulbecco's Modified Eagle Medium (DMEM) containing 10% FBS (FBS, HyClone, USA) in a humid environment at 37 °C with 5% CO2. LY294002 was purchased from Selleck (Selleck Chem, Houston, USA) and was dissolved in Dimethyl Sulphoxide (DMSO). DMSO was used for control.

### Cell transfection

eIF6, negative control (NC), sh-NC and sh-eIF6 (sh-eIF6-1, sh-eIF6-2) were all purchased from GemmaPharma (Suzhou,China). According to the manufacturer's protocol, plasmids were transfected into HN4 and HN6 cells using Lipofectamine 2000 (Invitrogen, Carlsbad, USA). The transfected cells were cultured in a complete medium for at least 48 h before performing the next experiment.

### Western blot

Total protein was lysed with lysis buffer (Beyotime, China) containing phosphatase inhibitor and protease inhibitor cocktails. Utilizing Coomassie Brilliant Blue as the standard, protein lysate was quantified with the bovine serum albumin (BSA). The total proteins were loaded into SDS–polyacrylamide gels and then transferred to polyvinylidene difluoride (PVDF) membranes (Millipore) with 5% BSA at room temperature for 2 h. Next, the membrane was incubated with primary antibodies (diluted 1:1000) specific for eIF6 (Abcam), E-cadherin, Vimentin, N-cadherin, AKT, p-AKT, and PI3K (CST), EGFR, p-EGFR, and β-actin (Bioworld, China), ZEB1 and ZEB2 (Proteintech, USA) and incubated overnight at 4℃. Samples were then washed three times with PBST for 10 min and incubated with anti-goat IgG HRP-conjugated secondary antibodies (Zhongshan Goldenbridge Bio, China) for 1 h at room temperature. Finally, the immunoreactive bands were detected by Immobilon Western Chemiluminescent HRP Substrate (Millipore) and visualized with the ImageQuantLAS4000 mini imaging system (General Electric). ImageJ software was used for gray value analysis and β-actin was used as an internal control. Each experiment was independently repeated three times and quantitatively analyzed.

### RNA extraction and quantitative reverse transcription PCR (qRT-PCR)

According to the reagent instructions, total RNA was obtained using TRIzol reagent (Invitrogen) and then reverted to cDNA using 5 × PrimeScript RT Master Mix (TaKaRa) after 15 min at 37℃ and 5 s at 85℃. Quantitative Real-Time PCR in a 7900HT Real-Time PCR System (Applied Biosystems). The RNA levels of eIF6 and GAPDH were determined with the following primers: eIF6: F: 5′- CCGACCAGGTGCTAGTAGGAA-3′, R: 5′- CAGAAGGCACACCAGTCATTC-3'; AKT: F: 5′- AGCGACGTGGCTATTGTGAAG-3′, R: 5′- GCCATCATTCTTGAGGAGGAAGT-3′; EGFR:F: 5′- AAAGTTAAAATTCCCGTCATCAG-3′, R: 5′-TCACGTA GGCTTCATCGAGATTTC-3′; PIK3CA:F: 5′-CCACGACCATCATCAGGTGAA-3′, R: 5′-CCTCACGGAGGCATTCTAAAGT-3′; PIK3CB: F: 5′-TATTTGGACTTTGCGACAAGACT-3′, R: 5′-TCGAACGTACTGGTCTGGATAG-3′; GAPDH: F: 5′-GAAGGTGAAGGTCGGAGT C-3′, R: 5′-GAGATGGTGATGGGATTTC − 3'. The result was quantified by the delta-delta Ct method to quantify the relative gene expression. The average expression of each gene was normalized to the geometric mean of GAPDH.

### Cell viability and colony formation assay

For cell viability experiment, cells were seeded in a 96-well plate at a density of 2000 cells per well and cultured at 37℃ for 0–7 days. After each time point, 10% CCK-8 reaction solution (DOJINDO, Japan) was added to each well and incubated for another 2 h at 37 °C medium to culture the cells to be measured for 2 h. The absorbance was quantified on a spectrophotometer microplate reader (Multiskan MK3, Thermo) with 450 nm wavelength. Eight experiments were independently conducted every day.

For the colony formation experiment, cells were cultured in 60-mm dish (Corning) with 2000 cells per dish for 14 days. Cells were then fixed with 4% paraformaldehyde (PFA), stained with crystal violet, and analyzed by microscopy.

### Transwell assay

Cell invasion ability was analyzed through Transwell filters (8 mm pore size; Millipore) coated with 50 mL Matrigel Basement Membrane Matrix (BD Biosciences). The cells (40,000 cells) were seeded in the upper chamber containing 200μL of serum-free medium, while 800μL of 10% serum medium was placed in the lower chamber. After indicated time point, cells in the upper chamber were fixed with 4% PFA for 30 min, stained with crystal violet, and analyzed under the microscope (ZEISS, Germany).

### Wound healing assays

The cell migration was performed using a wound-healing assay. Briefly, cells were seeded in a 6-well plate at 2000 cells per well for 24 h. A line was then drawn using a marker on the bottom of the dish, after which a sterile 10 µL pipet tip was used to scratch three separate wounds through the cells, moving perpendicular to the line. The cells were gently rinsed twice with PBS to remove floating cells and incubated in cultured in a serum-free medium. Images of the scratches were taken by using an optical microscope (Leica, Germany) at × 10 magnification at indicated time of incubation, and the healing area was analyzed with ImageJ software (Wayne Rasband National Institutes of Health, USA).

### Immunofluorescence staining

HN6 transfected cells were cultured on the sterile glass-coverslips in 24-well plates for 12 h. Cells were then fixed with 4% PFA and permeabilized with 1% Triton and blocked with goat serum for 30 min. In the shaded condition, the cells were incubated with the eIF6 (diluted 1:100, Abcam) or AKT (diluted 1:100, CST) or p-AKT (diluted 1:200, CST) antibodies at 4℃ overnight and then stained with goat anti-rabbit IgG antibody Cy3 (Proteintech, China) for 1 h at 37℃. After staining with DAPI (Sigma, St Louis, MO), cells were analyzed using fluorescence microscopy (ZEISS, Germany).

### Subcutaneous tumor model

Twenty male BALB/c athymic nude mice (five-week-old) were purchased from the Animal Core Facility of Nanjing Medical University (Nanjing, China). All the animals were housed in an environment with a temperature of 22 ± 1 ºC, relative humidity of 50 ± 1%, and a light/dark cycle of 12/12 h. All animal studies (including the mice euthanasia procedure) were done in compliance with the regulations and guidelines of Nanjing Medical institutional animal care and conducted according to the AAALAC and the IACUC guidelines.

Animals were randomly divided into 4 groups (5 mice per group): NC, eIF6, sh-NC, and sh-eIF6. Stably transfected HN6 cells resuspended in 50% matrigel were subcutaneously injected into the nude mice flank (1 × 10^7^ cells/100 μL). The xenograft tumor size was checked every three days and measured with a vernier caliper. The formula that was used to measure the tumor volume was: *[volume* = *(length* × *width*^*2*^*)/2]*. Twenty days after the injection, the nude mice were executed, and the tumor tissues were dissected out, imaged, and weighed up.

### Statistical analysis

Statistical analysis is processed using graphing software GraphPad Prism version 8.0.1 (Graph Pad Software Inc., La Jolla, CA, USA). All experiments are repeated at least 3 times. The data index of each experiment represents the mean ± SD from 3 independent experiments. According to the normality of the sample, we utilized the Student's t-test or the Wilcoxon signed-rank test to calculate the statistical significance of experimental data. All P values represent statistically significant two-sided tests (*P < 0.05, **P < 0.01, ***P < 0.001).

## Results

### Cytoplasmic eIF6 is overexpressed in OSCC and is associated with poor prognosis

**Fig. 1:**
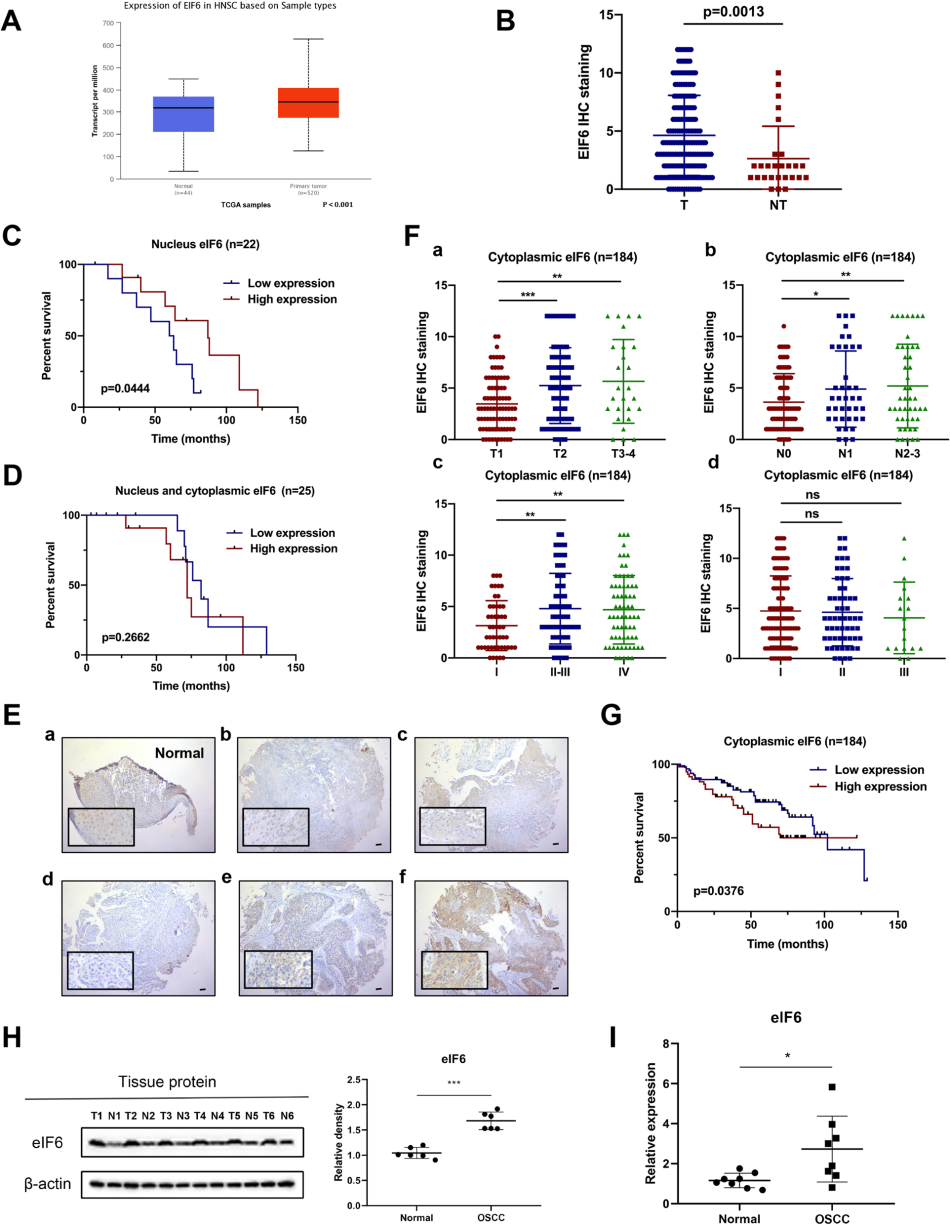
Cytoplasmic eIF6 is overexpressed in OSCC and is associated with poor prognosis. (**A**) Expression of eIF6 in HNSCC patients based on the TCGA database. (**B**) Pathological scores on eIF6 were assessed in tumor (T) and non-tumor (NT) tissues. (**C**) Low expression of nucleus eIF6 was associated with poor prognosis (n = 22, p < 0.005). (**D**) No prognostic difference was observed in the nucleus and cytoplasmic co-expression of EIF6 between the low- and high-expression group. (**E**) Representative IHC micrographs of tumor microarrays sections stained with eIF6. (**a**) non-tumor tissue; (**b**) tumor tissue with eIF6 nucleus staining; (**c**) tumor tissue with eIF6 nucleus and cytoplasmic co-expression; (**d**) The weak, (**e**) moderate and (**f**) strong level of eIF6 staining were demonstrated. (**F**) The expression of eIF6 in OSCC patients with different T stage, N stage, clinical stage and pathological stage. (**G**) The survival curve of cytoplasmic eIF6 expression on prognosis. (**H**) The expression level of eIF6 protein in six tumor and adjacent non-tumor OSCC tissues. Density analysis of eIF6 signal with β-actin as the internal control. (**I**) The eIF6 mRNA expression in eight tumor and adjacent non-tumor OSCC tissues. In OSCC, the protein or mRNA expression of eIF6 is significantly higher than that of non-tumor tissues. All error bar values represent the SD. Scale bar, 100 μm. *P < 0.05, **P < 0.01, ***P < 0.001

To explore how eif6 is expressed and whether it has a role in OSCC, we conducted differential expression analysis in the TCGA database by an interactive web portal, UALCAN (http://ualcan.path.uab.edu) [30]. The results showed a marked difference in eIF6 expression between the tumor and non-tumor groups, and the difference was statistically significant (Fig. 1A). Then we performed a tissue microarray analysis, including 206 tumor tissues and 27 non-tumor tissues. Among 206 tissues, 22 tissues showed a nucleus expression of eIF6. In 25 tumor tissues, eIF6 was expressed in both nucleus and cytoplasm. Cytoplasmic-specific expressing eIF6 was observed in 184 tissue sections, which were further analyzed by clinicopathological features (Table 1). In normal oral mucosa, eIF6 was mostly confined to the nucleus with lower expression compared with tumor tissues (Fig. 1B,E). We also found that low expression of nucleus eIF6 was associated with poor prognosis, indicating the positive role of the nucleus eIF6 in OSCC (Fig. 1C). However, there was no prognostic difference in the nucleus and cytoplasmic co-expression of EIF6 between the low- and high-expression group (Fig. 1D). Notably, in tumor tissues, eIF6 was mostly diffused in the cytoplasm (Fig. 1E). High cytoplasmic eIF6 staining was significantly associated with advanced tumor size, lymph node metastasis, and clinical stage (Fig. 1F and Table 1). No significant difference was shown in the pathological stage. The Kaplan–Meier curves indicated that increased cytoplasmic eIF6 expression was related to lower overall survival in OSCC patients (Fig. 1G).

We then collected 8 OSCC tumor tissues and the same amount of normal tissues from the clinic. Through Western blot (n = 6) and qRT-PCR (n = 8) analysis, we found that eIF6 was overexpressed in OSCC than in normal tissues (Fig. 1H–I). These results indicated that the presence of eIF6 has an essential role in OSCC.

### eIF6 enhances OSCC proliferation in vitro* and *in vivo

**Fig. 2:**
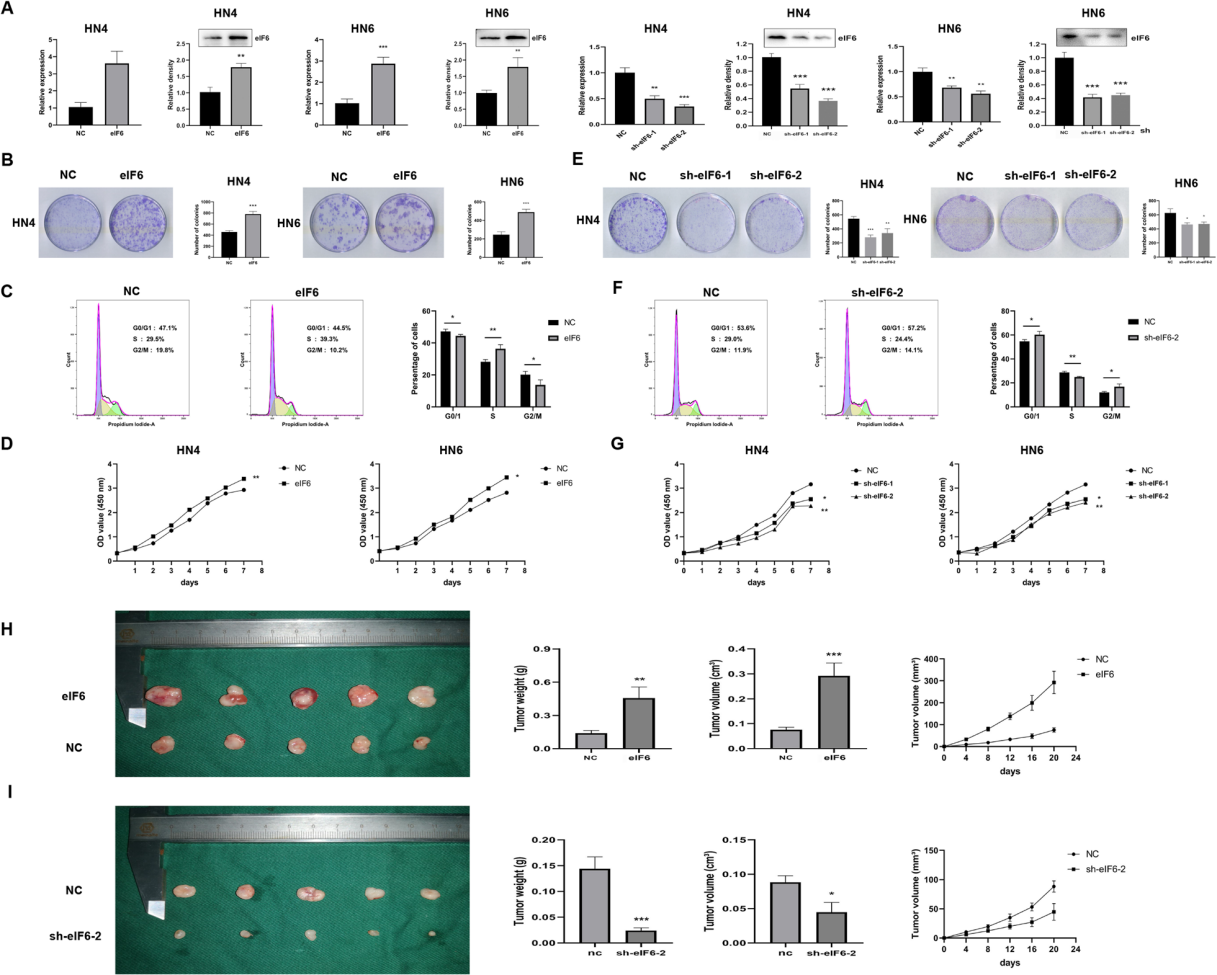
eIF6 enhances OSCC proliferation in vitro* and *in vivo. (**A**) Efficiency of eIF6 overexpression and knock-down was assessed by qRT-PCR and Western blotting analysis. (**B**) Colony formation assay in eIF6 over-expressed HN4 and HN6 cells. (**C**) Flow cytometry analysis in eIF6 over-expressed HN6 cells. (**D**) CCK8 assay of eIF6 overexpression was investigated. eIF6 greatly promoted cell proliferation. (**E**–**F**) eIF6 knockdown resulted in anti-proliferative effect in colony formation and flow cytometry analysis. (**G**) CCK-8 assays confirmed the anti-proliferative effect of eIF6 in OSCC cell lines. (**H-I**) eIF6 over-expression promoted tumor growth and depletion of eIF6 inhibited tumor progression in vivo. The tumors dissected from mice were presented. Tumor weight and tumor volume were demonstrated on the right respectively. *P < 0.05, **P < 0.01, ***P < 0.001

In order to determine the function of eIF6 in tumor progression, we performed gain-of-function or loss-of-function assays in HN4 and HN6 OSCC cell lines. The efficiency of infection was confirmed by qRT- PCR. Marked increase or depletion of eIF6 expression level was observed in Fig. 2A. Through the above research, we began to explore how eIF6 affects the phenotype of OSCC cells. Transfection with eIF6 formed larger colonies and improved cell proliferation ability (Fig. 2B). Flow cytometry analysis confirmed the proliferative tendency in eIF6 over-expression group (Fig. 2C). Compared with the control group, the up-regulation of eIF6 resulted in a decrease in the percentage of cells in the G0/G1 phase and an increase in the S phase, which showed that cell division was in an active stage at this time. Moreover, the CCK8 assay of eIF6 overexpression was investigated with HN4 and HN6 cells (Fig. 2D). The rate of cell growth and proliferation significantly increased compared with control. Furthermore, the inhibition of eIF6 formed fewer colonies, and reduced cell proliferation (Fig. 2E). The flow cytometry indicated that, lowering eIF6 led to an increase in the percentage of G0/1 phase and a decrease in the percentage of S phase. The eIF6 knockdown might prevent the transition from G1 to S phase, and cell division is arrested in G0/G1 phase (Fig. 2F). Similarly, CCK-8 assays confirmed the anti-proliferative effect of eIF6 in OSCC cell lines (Fig. 2G).

Next, we analyzed the effects of eIF6 in vivo. The NC, eIF6, sh-NC and sh-eIF6 encoding vectors were transfected into HN6 cell lines, respectively. Then, the transformed HN6 cell lines were subcutaneously inoculated in BALB/C nude mice in 4 groups. The tumors were dissected 20 days after tumor formation. As shown in Fig. 2H, cells overexpressing eIF6 grew faster (higher tumor weight and volume) than the cells expressing eIF6 encoding vectors or control vectors. By contrary, eIF6 depletion attenuated tumor formation in vivo (Fig. 2I).

### High expression of eIF6 can induce epithelial-mesenchymal transition, while knockdown of eIF6 reversed this process

**Fig. 3:**
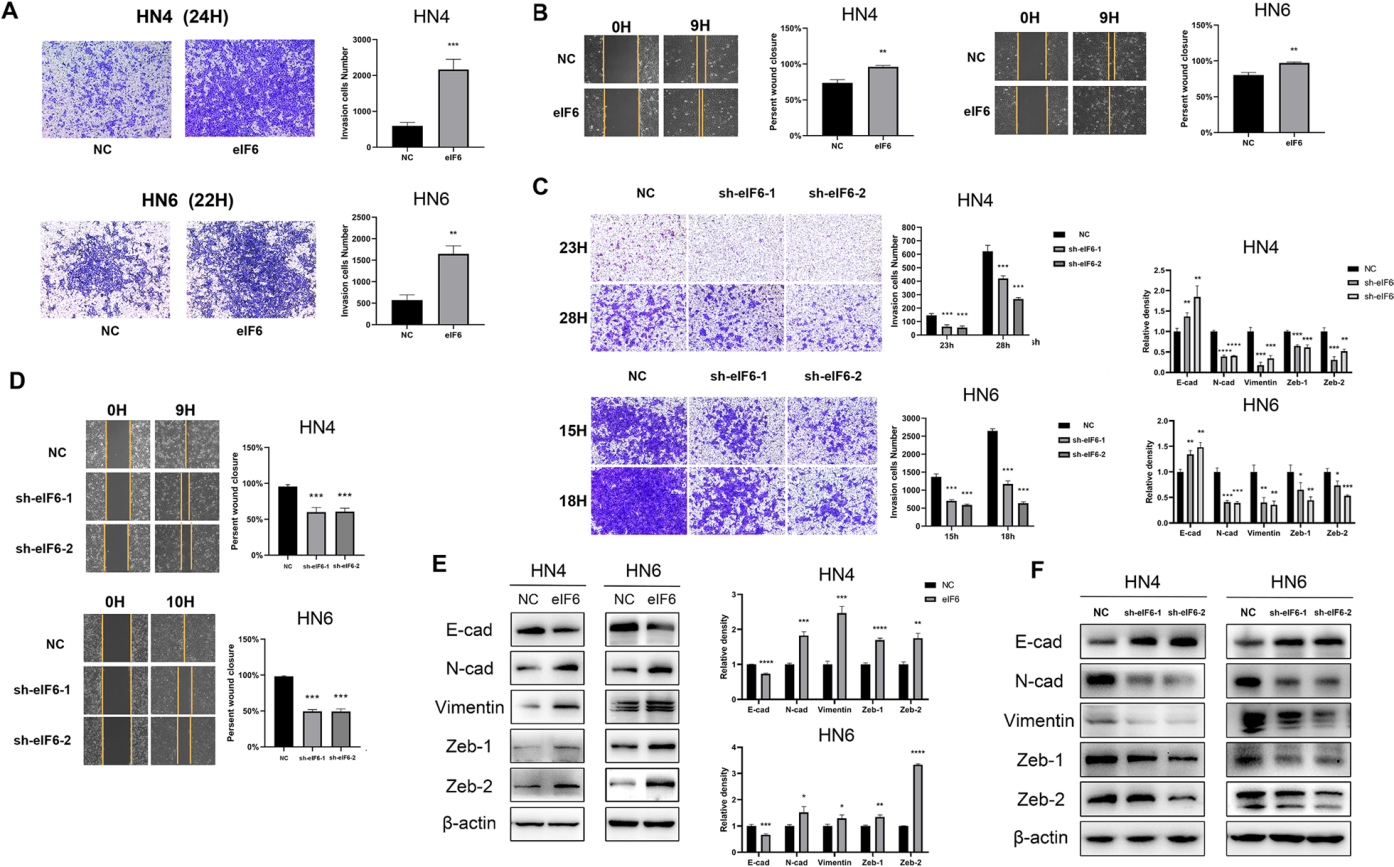
High expression of eIF6 can induce epithelial-mesenchymal transition, while knockdown of eIF6 reversed this process. (**A**) Transwell experiment was performed in HN4 and HN6 eIF6-transfected or NC-transfected cells (50 × magnification). eIF6 enhanced invasive capacity of OSCC cells. (**B**) The scratch assay was performed in HN4 and HN6 eIF6-transfected cells. (**C**) Transwell experiment was performed in HN4 and HN6 sh-eIF6-transfected or sh-NC-transfected cells (50 × magnification). (**D**) Wound healing experiment was demonstrated in sh-eIF6-transfected or sh-NC-transfected OSCC cells. (**E**) The relationship between high expression of eIF6 and EMT-related markers in HN4 and HN6 cells by western blot analysis. (**F**) Western blot analysis showed the effect of eIF6 knockdown on EMT in HN4 cells. All error bar values represent the SD. *P < 0.05, **P < 0.01, ***P < 0.001

Generally, the aggressiveness and metastasis of OSCC are the main reasons for the poor prognosis [31]. In order to develop metastasis, tumor cells need to acquire aggressive activity. Epithelial-mesenchymal transition (EMT) can induce cells to change their morphology, lose their polarity, and increase invasiveness. Therefore, it is easier for tumor cells to transfer from the original environment to distant places[32]. Translation factors, such as the PERK/eIF2/ATF4 signaling pathway, have been involved in EMT in pancreatic cancer cells[33]. To explore whether eIF6 can regulate EMT in OSCC, we stably transfected control and eIF6 encoding vectors into HN4 and HN6 cell lines. Overexpression of eIF6 significantly strengthened the invasion ability (Fig. 3A) and cell migration compared to the control group (Fig. 3B) in HN4 and HN6 cells. Conversely, the inhibition of eIF6 inhibited cell invasion and migration (Fig. 3C,D). Subsequently, we studied the relationship between high expression of eIF6 and EMT in HN4 and HN6 cells. We measured the expression of EMT-related markers through Western blot analysis (Fig. 3E). High expression of eIF6 down-regulated the epithelial markers E-cadherin and upregulated mesenchymal markers N-cadherin and Vimentin, as well as transcription factors Zeb1 and Zeb2. These results revealed that over-expression of eIF6 induced EMT in HN4 and HN6 cells. Contrary, the knockdown of eIF6 reversed this process, i.e., E-cadherin protein content increased, while N-cadherin, Vimentin, Zeb1, and Zeb2 significantly decreased (Fig. 3F). This result showed to some extent that knocking down eIF6 can reverse the EMT phenomenon in OSCC cells.

### eIF6 effect is related to the AKT signaling pathway

**Fig. 4:**
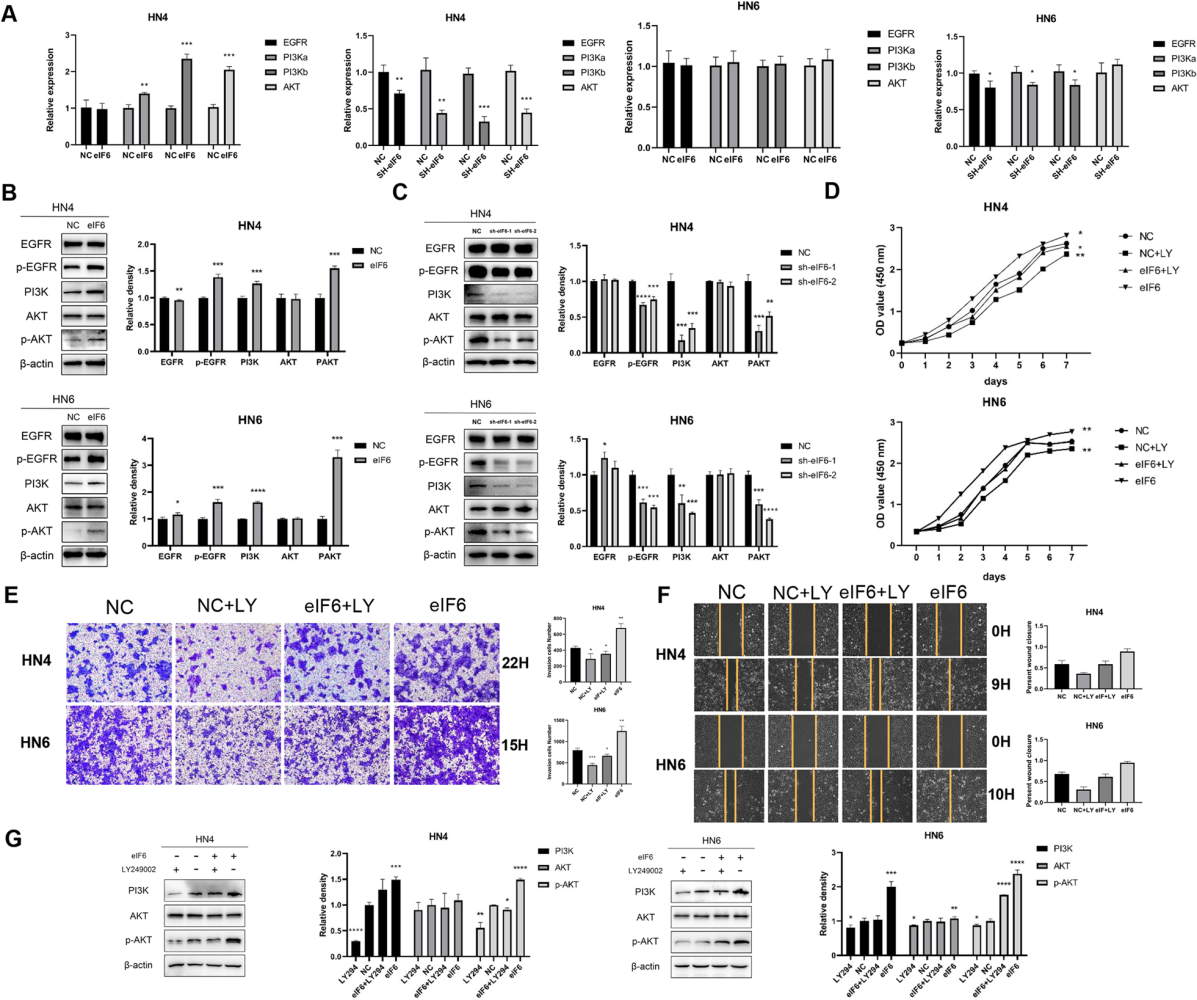
eIF6 effect is related to the AKT signaling pathway. (**A**) mRNA changes in PIK3CA, PIK3CB, AKT and EGFR upon upregulation or downregulation of eIF6 in OSCC cells. (**B**) Western blot analysis of EGFR-AKT pathway-related markers after the increase of eIF6 in HN4 and HN6 cells. (**C**) Inhibition of eIF6 caused a visible reduction in p-EGFR, PI3K, and p-AKT protein expression. (**D**–**F**) LY294002 decreased the oncogenic phenotype of eIF6 by depressing proliferative (**D**), invasive (**E**) and migrative (**F**) ability. (**G**) Western blotting analysis in cells transfected with eIF6 or NC plasmids with or without the treatment of LY294002. All error bar values represent the SD. *P < 0.05, **P < 0.01, ***P < 0.001

Previous experiments have shown that eIF6 may be related to the AKT signaling pathway [34]. To further explore the relationship between eIF6 and AKT signaling pathway in OSCC cells, we measured the cells’ mRNA changes associated with the AKT signaling pathway when eIF6 was amplified. In cells overexpressing eIF6, EGFR, phosphatidylinositol 3-kinase catalytic subunit alpha (PIK3CA), PIK3CB, and AKT were slightly increased (Fig. 4A); this process was reversed when knocking down eIF6 (Fig. 4A). Next, we performed Western blot and found that the high expression of eIF6 had protein effects on the AKT pathway-related markers (Fig. 4B). In HN4 and HN6 cells, the results showed that the expressions of phospho-EGFR (p-EGFR), PIK3CA, PIK3CB, and p-AKT were higher than those of the control group, but the changes of EGFR and AKT were not noticeable. Furthermore, the inhibition of eIF6 in both HN4 and HN6 cells caused a visible reduction in p-EGFR, PI3K, and p-AKT protein expression (Fig. 4C). These data indicated that the decrease in eIF6 expression could inhibit initially activated AKT pathway. To prove that cells overexpressing eIF6 do activate AKT signaling, we inhibited the AKT signaling pathway. LY294002, a PI3K inhibitor, was used in HN4 and HN6 cell lines overexpressing eIF6. Notably, LY294002 decreased the oncogenic phenotype of eIF6 by depressing proliferative, invasive and migrative ability (Fig. 4D–F). Furthermore, LY294002 significantly down-regulated the protein expression of PI3K and p-AKT in the cells transfected with eIF6 (Fig. 4G), suggesting a crosstalk between eIF6 and PI3K-AKT pathway.

### eIF6 participates in the activation of AKT in the cytoplasm

**Fig. 5:**
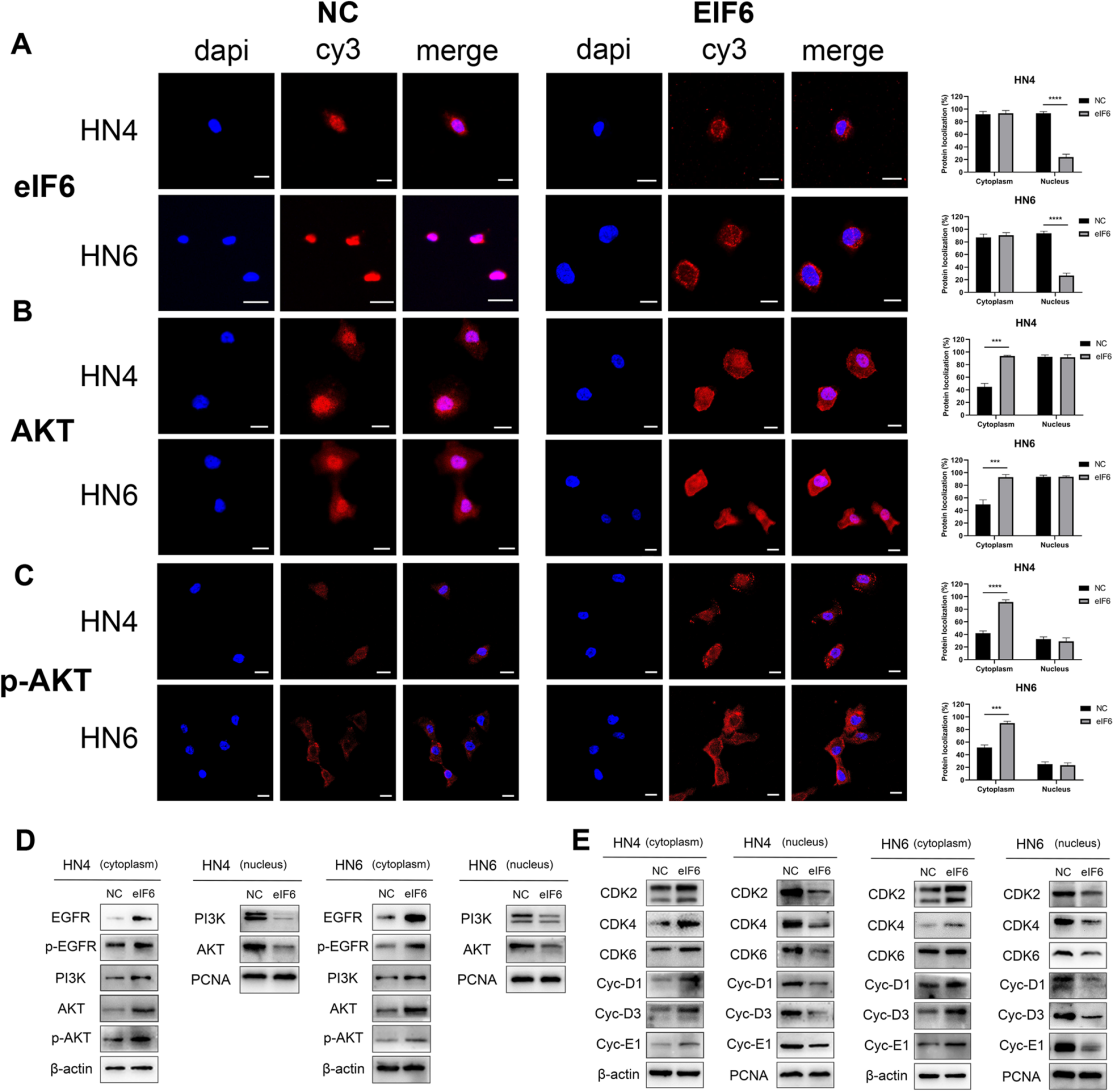
eIF6 participates in the activation of AKT in the cytoplasm. (**A**) Confocal microscopy showed that eIF6 was mainly expressed in the cytoplasm upon eIF6 amplification compared with control. (eIF6, red; DAPI depicts nuclei, blue). (**B,C**) Increased immunostaining of AKT (**B**) and p-AKT (**C**) in the cytoplasm was shown upon ectopic expression of eIF6. (**D**) eIF6 augmentation resulted in the upregulation of cytoplasmic expression of eIF6, AKT and p-AKT, while nucleus expression of eIF6, AKT and p-AKT was reduced after transfection. (**E**) Cell-cycle related proteins were further analyzed in the subcellular fractions. All error bar values represent the SD. Scale bar, 20 μm. *P < 0.05, **P < 0.01, ***P < 0.001

In our previous study, we have discovered the oncogenic role of cytoplasmic eIF6 in tumor tissues by microarray analysis (Fig. 1G). We then investigated the physical localization of eIF6 in OSCC cells. Confocal microscopy showed that eIF6 was mainly expressed in the cytoplasm upon eIF6 amplification compared with control cells where eIF6 was demonstrated in the nucleus (Fig. 5A). AKT and p-AKT expression levels were then examined by immunofluorescence. Over-expression of eIF6 resulted in an activation of AKT pathway with an increased immunostaining of AKT and p-AKT in the cytoplasm (Fig. 5B,C). Decreased nuclear translocation of AKT and increased phosphorylation of AKT were detected in eIF6-over-expressing cells; however, cells in the control group exhibited the opposite pattern of nuclear translocation and phosphorylation. Protein separation techniques were utilized to determine the protein level of subcellular fractions. Western blotting was performed to assess the expression of eIF6 and AKT pathway proteins in cytoplasmic and nuclear extracts from eIF6-over-expressed or control cells. Cytoplasmic expression of eIF6, AKT and p-AKT in eIF6-over-expression group was increased compared with control (Fig. 5D). Meanwhile, eIF6 inhibition displayed an opposite effect (data not shown). We then analyzed cell-cycle related proteins by performing Western blotting. We found that amplification of eIF6 resulted in an up-regulation of cytoplasmic CDK (CDK2, 4) and cyclin (cyclin D1, cyclin D3 and cyclin E1) proteins in both HN4 and HN6 cell lines (Fig. 5E). These results suggested that functional eIF6 was retained in the cytoplasm and regulated cell-cycle checkpoints by activating AKT pathway.

### eIF6 physically interacts with AKT

**Fig. 6:**
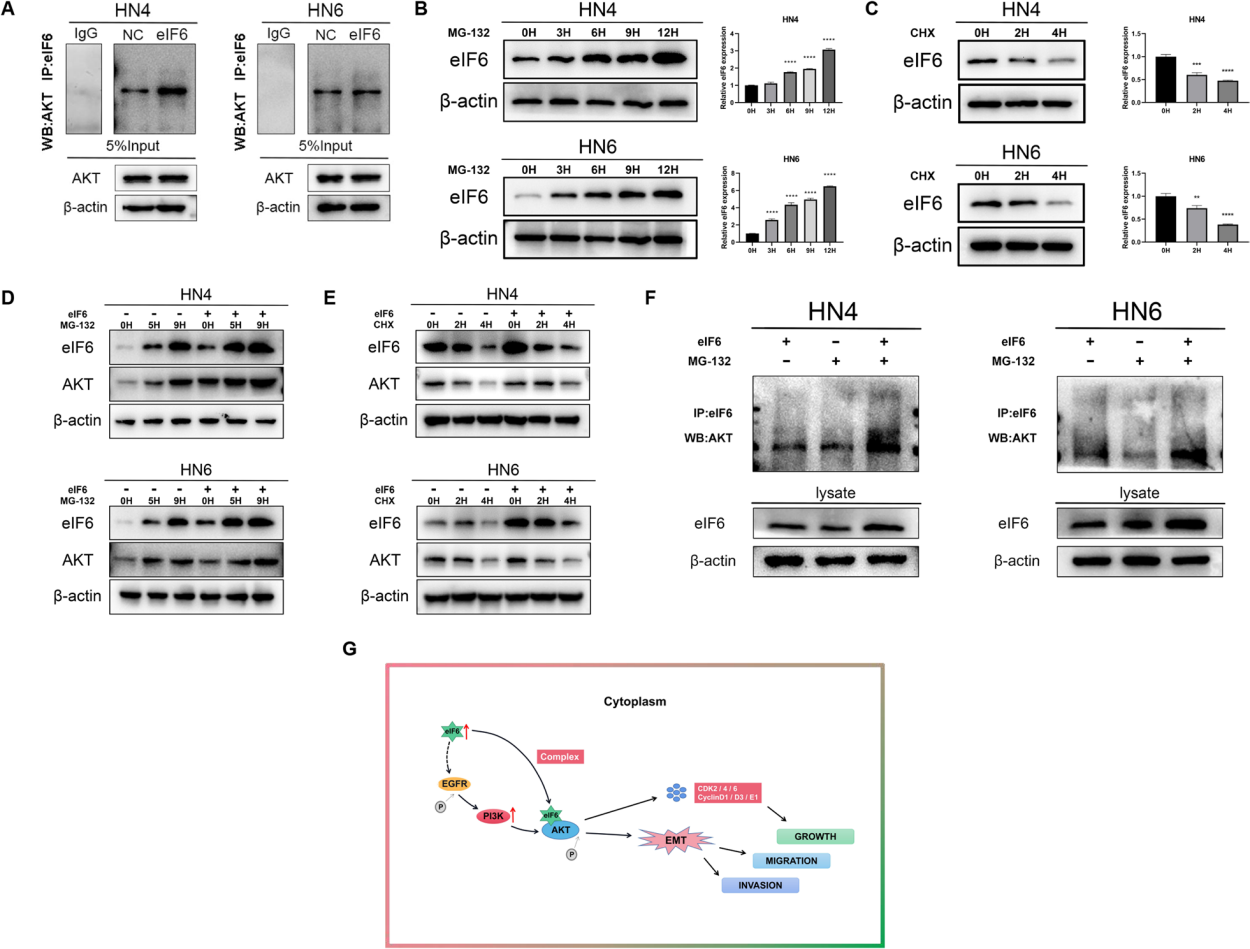
eIF6 physically interacts with AKT. (A) Immunoprecipitation assay between endogenous eIF6 and AKT. (**B**) The protein levels of eIF6 and AKT were detected in cells treated with MG132 (10 μM). (**C**) The protein levels of eIF6 and AKT were detected in cells treated with CHX (20 μM). (**D**) Cells over-expressing eIF6 were treated with or without MG132, and the eIF6 and AKT protein levels were assessed by Western blotting. (**E**) CHX was utilized in cells transformed with eIF6 or NC plasmids, and the protein expression levels of eIF6 and AKT were displayed. (**F**) Co-IP experiment showed MG132 promoted the binding extent between eIF6 and AKT. The cells in eIF6 over-expression group were treated with or without MG132. Cell lysates were prepared and subjected to immunoprecipitation with anti-eIF6 antibody. The level of AKT was detected by western blotting analysis. (**G**) Graphical abstract demonstrated the regulation of eIF6/AKT axis in the cytoplasm

To further determine whether there was a direct interaction between endogenous eIF6 and AKT protein, we investigated the protein–protein interaction by immunoprecipitation assay in OSCC cells (Fig. 6A). Endogenous eIF6 was co-immunoprecipitated with endogenous AKT in the cytoplasm in both HN4 and HN6 cells. Cells were then treated with MG132 (10 μM) for the indicated times, and then the levels of eIF6 and AKT were detected. MG132 enhanced both eIF6 and AKT protein levels in a time-dependent manner (Fig. 6B). Opposite effect was observed in CHX-treated OSCC cells (Fig. 6C). Then cells over-expressing eIF6 were treated with or without MG132, and the eIF6 and AKT protein levels were assessed by Western blotting (Fig. 6D). Ectopic expression of eIF6 increased the amount of AKT when exposed to MG132. CHX treatment resulted in adverse impact (Fig. 6E). Furthermore, with the exposure of MG132, ectopic expression of eIF6 increased the amount of co-immunoprecipitated AKT (Fig. 6F). These data indicated that cytoplasmic eIF6 mediated the AKT binding, thereby facilitating AKT pathway activation.

## Discussion

eIF6 is the primary regulator of translation and tumor progression in vivo. In the cytoplasm, it affects the assembly of 80S by binding to 60S ribosomes [35]. eIF6 is also believed to be a cancer-related biomarker and a potential therapeutic target for malignant tumors [36]. Changes in eIF6 have been found in some malignant tumors. Its high expression was found in HNSCC [37]. In this study, we investigated the regulation of eIF6 on OSCC and its possible mechanism. We performed a microarray analysis of tumor tissue samples from OSCC patients and noticed that eIF6 was highly expressed in cancer tissues compared to normal tissue. Moreover, cytoplasmic eIF6 was significantly correlated with the tumor size, lymph node metastasis and clinical stage of patients. We also observed that high expression of cytoplasmic eIF6 indicated a poor prognosis in the survival curve. These results suggested that eIF6 could be a diagnostic and prognostic marker for OSCC.

Here, we used plasmids over-expressing or depleting of eIF6 to examine its role in OSCC. Results showed that high expression of eIF6 could increase the biological behavior of OSCC, including cell viability, proliferation, invasion, and migration. Knockdown of eIF6 can significantly inhibit this cancer-promoting effect. Thus, we hypothesized that it is the abnormal accumulation of eIF6 in cells that leads to the continuous deepening of the malignancy of OSCC.

Epidermal growth factor receptor (EGFR) belongs to the receptor tyrosine kinases, which is highly expressed in most OSCC and is associated with poor prognosis [38]. The EGFR/phosphoinositide 3-kinase (PI3K)/AKT pathway has an important role in cell physiology, activating mTOR to participate in a wide range of life activities, including proliferation, growth, movement, and metabolism [39]. Studies have suggested that the translation behavior of eIF6 is not regulated downstream of the mTOR signaling pathway. Specifically, the activation of eIF6 is derived from phosphorylation caused by the protein kinase C system (PKC), not mTORC1[40]. This means that regulating eIF6 may effectively avoid the emergence of mTOR-resistance. Nonetheless, the mechanism underlying eIF6 action is still not clear. Some scholars have found significantly enriched eIF6 expression and PI3K/AKT/mTOR signals in colorectal adenomas [41]. As the classical upstream regulator of AKT, EGFR also has a regulatory connection with the eukaryotic translation factor [42]. Our study suggested that eIF6 amplification could increase p-EGFR, PI3K, and p-AKT, but not EGFR and AKT. At the same time, knocking down eIF6 showed an inhibitory effect on the corresponding indicators. These results suggested that eIF6 can stimulate the PI3K/AKT cascade, which is consistent with the tumor cell phenotype after eIF6 amplification. Crucially, the PI3K/AKT signaling pathway may also have an essential role in eIF6-mediated EMT.

To confirm that eIF6 is indeed involved in the PI3K/AKT cascade reaction, LY294002, an inhibitor of PI3K, was used to further process the cells. It has been clinically proven that LY294002 has significant effects on inhibiting tumor growth and treatment [43]. We did not observe complete inhibition of p-AKT, which suggested that there may still be other ways to activate the AKT pathway. But this also confirms that even with higher eIF6 levels, PI3K inhibition can still directly block AKT regulated by eIF6. Mechanically, we found that eIF6 could directly bind with AKT in the cytoplasm, indicating the physical interaction between two proteins.

Moreover, we also found that after eIF6 was amplified, the subcellular localization of eIF6 was significantly changed. It appeared in large numbers in the cytoplasm but rarely accumulated in the nucleus. This indicated that accelerating the nuclear transport of eIF6 may be closely related to the invasion and metastasis of cancer. Currently, researchers generally believed that the transportation of eIF6 had to go through a series of complicated processes. In the nucleolus, eIF6 was bound to the immature large ribosomal subunit (pre-60S) and other regulatory proteins. With the mature process of 60S subunit, eIF6 was exported to the cytoplasm [13]. Then PKC (protein kinase C) was recruited by RACK1 (Receptor For Activated C Kinase 1) to the 40S subunit and phosphorylated eIF6, causing eIF6 release from 60S subunit. [44, 45]. The correct combination of 40S and 60S ribosomal subunit ensured the subsequent translation process. Therefore, phosphorylation might modulate the localization of eIF6. Several eIF6 phosphorylation phenomena had also been reported. During serum starvation, eIF6 accumulated in the cytoplasm, and the change in localization depended on the phosphorylation of GSK3[46]. The Ca2 + /calmodulin-dependent protein phosphatase calcineurin could mediate the dephosphorylation of eIF6, which promoted the return of eIF6 to the nucleolus[9]. In addition, studies found that malignant pleural mesothelioma tumors contained high levels of hyperphosphorylated eIF6, and dephosphorylated eIF6 significantly reduced the growth and metastasis of tumor cells[23]. Through the understanding of the above process, we believed that the cells overexpressing eIF6 was in a state of abnormal proliferation, and the high level of proliferation meant this excessive demand for translation. On the one hand, due to the acceleration of eIF6 nuclear export. On the other hand, the efficiency of import nucleus was greatly reduced, which might be due to abnormal phosphorylation or dephosphorylation of eIF6. The balance of eIF6 between nucleolus and cytoplasm might be broken. Therefore, eIF6 was largely retained in the cytoplasm, but almost not in the nucleus. In general, a large increase in cytoplasmic eIF6 meant higher proliferation invasive capacity. The transformation of eIF6 from cytoplasm to nucleus may indicate a new perspective for the treatment of this type of cancer. Further experiments are needed to explore the relationship between phosphorylated eIF6 level and AKT phosphorylation.

## Conclusion

This was a significant report on the mechanism of eIF6 in OSCC (Fig. 6G). Our study explained the underlying mechanism of eIF6-related phenotypes and emphasized the critical part of the AKT signaling pathway in eIF6-mediated cancer. In the clinic, eIF6 is expected to become an essential criterion for diagnosing the tumor condition and judging the prognosis.

## Supplementary Information

**Additional file 1.** **Table S1:** Clinical features of 8 patients with OSCC.

## Data Availability

All data generated or analyzed during this study are included in this published article.

## Abbreviations

OSCC: Oral squamous cell carcinoma
eIF6: Eukaryotic translation initiation factor 6
IHC: Immunohistochemical staining
IF: Immunofluorescence
co-IP: Co-immunoprecipitation
EMT: Epithelial-mesenchymal transformation
ZEB1: Zinc-finger E-box binding homeobox 1
ZEB2: Zinc finger E-box binding homeobox 2)
PERK-eIF2α: Protein kinase RNA-like ER kinase-eIF2
Rac1: Rho-family small GTPases
PIK3CA: Phosphatidylinositol 3-kinase catalytic subunit alpha
mTORC1: MTOR complex 1
EGFR: Epidermal growth factor receptor
PKC: Protein kinase C system
