# Supplementary material for: Cytoplasmic eIF6 promotes OSCC malignant behavior through AKT pathway
Source: Cell Commun Signal. 2021 Dec 18;19:121. doi: 10.1186/s12964-021-00800-4 (PMC8684100; doi:10.1186/s12964-021-00800-4)
Supplement: Supplementary file 2 — Additional file 1. Table S1: Clinical features of 8 patients with OSCC. [file 12964_2021_800_MOESM2_ESM.doc]

Table S1. Clinical features of 8 patients with OSCC

| No. | Age | Sex | Location | TNM | Differentiation |
| --- | --- | --- | --- | --- | --- |
| 1 | 69 | M | Tongue | T1N0M0 | Well |
| 2 | 53 | M | Floor of mouth | T2N0M0 | Poor |
| 3 | 76 | M | Buccal | T2N2bM0 | Moderate |
| 4 | 60 | F | Floor of mouth | T2N0M0 | Moderate to poor |
| 5 | 75 | M | Gingiva | T2N0M0 | Well |
| 6 | 61 | F | Buccal | T2N2bM0 | Moderate |
| 7 | 62 | M | Tongue | T1N0M0 | Moderate to poor |
| 8 | 56 | F | Tongue | T2N1M0 | Moderate |

OSCC oral squamous cell carcinoma, F female, M male; TNM classification and tumor stage were determined by the Union for International Cancer Control (UICC)
